# Editorial: Updates on giant cell arteritis: pathogenesis, diagnosis and treatment, volume II

**DOI:** 10.3389/fmed.2024.1522736

**Published:** 2024-11-28

**Authors:** Andreas P. Diamantopoulos, Stavros Chrysidis

**Affiliations:** ^1^Department of Infectious Diseases, Akershus University Hospital, Lørenskog, Norway; ^2^Department of Rheumatology, University Hospital of Southern Denmark, Esbjerg, Denmark

**Keywords:** GCA, imaging, outcomes, treatment, relapse/refractory

## Introduction: the complexity of GCA diagnosis and management

Giant Cell Arteritis (GCA) presents significant diagnostic and treatment challenges, given its systemic nature, primarily targeting large, and medium-sized arteries in patients over 50. Without timely intervention, GCA can cause devastating complications such as sight loss, stroke, and aortic aneurysms. Recent advancements in imaging technologies, classification criteria, and novel therapies have greatly improved our ability to diagnose and manage this complex disease. However, important questions remain regarding relapse monitoring and long-term treatment strategies.

This editorial presents the latest evidence published in this issue regarding diagnostic innovations, phenotypic variability, clinical management of relapses, and emerging steroid-sparing treatments.

## Temporal artery biopsy: still relevant despite advancements

The role of temporal artery biopsy (TAB), while debated, remains pivotal in diagnosing cranial GCA. Stamatis et al. underscore its continued importance despite the rise of imaging techniques such as ultrasound (US) and Positron Emission Tomography (PET). TAB has high specificity for cranial GCA, though its sensitivity can vary, particularly in patients with large-vessel GCA (LV-GCA). Specimen length, number of sections, and biopsy timing (preferably within 2 weeks of starting glucocorticoids) influence TAB's diagnostic yield.

Although imaging techniques have improved, Stamatis et al. underscore that TAB is essential for differentiating healed arteritis from age-related atherosclerosis, which can be diagnostically challenging. Thus, TAB continues to play a role in diagnosing cranial GCA, especially when imaging results are unclear.

## Imaging advances: expanding diagnostic possibilities

US has emerged as a non-invasive alternative to TAB for diagnosing GCA, mainly through the halo sign, which may indicate temporal artery inflammation. In a landmark study by Haaversen et al., ultrasound was critical for diagnosis and follow-up, though its sensitivity for relapse detection was only 61.2%. To improve monitoring, Haaversen et al. introduced the GCA Activity Score (GCAS), which integrates ultrasound, clinical symptoms, and inflammatory markers like CRP, proving helpful in detecting subclinical relapses. PET and Magnetic Resonance Imaging (MRI) have demonstrated value in identifying LV-GCA, where inflammation extends beyond cranial vessels. Gorlen et al. showed that PET is particularly effective for excluding malignancies in patients with suspected polymyalgia rheumatica (PMR) or GCA.

A retrospective cohort study by Andel et al. using the updated 2022 ACR/EULAR classification criteria found that mixed-GCA (cranial and large-vessel involvement) was the most common phenotype, supporting the use of advanced imaging to identify and classify GCA subtypes more effectively.

## Phenotypic diversity and clinical challenges

GCA presents with diverse clinical phenotypes, complicating the diagnostic process. The classic cranial GCA is characterized by headache, jaw claudication, and vision problems, while LV-GCA manifests more subtly, with systemic symptoms like fever and weight loss. Skaug et al. found that patients with non-cranial GCA experienced longer diagnostic delays than those with cranial involvement.

These findings suggest the importance of regularly imaging non-cranial arteries in suspected GCA cases, especially in younger patients, to avoid diagnostic delays. Given that non-cranial GCA patients are often underdiagnosed, prompt imaging can reduce the risk of complications such as aortic aneurysms and stroke, as supported by Ayo-Martin et al., who found that vertebral vasculitis is significantly associated with increased stroke risk.

## Relapses: a persistent challenge in GCA management

Relapses in GCA remain a primary clinical concern, with rates as high as 60.6%, as Haaversen et al. noted. Contrary to earlier beliefs, relapses occur at similar rates across cranial, large-vessel, and mixed subtypes, underscoring the need for consistent monitoring. The GCA Activity Score (GCAS), which combines clinical, biochemical, and imaging data, has emerged as an essential tool for identifying subclinical relapses that might otherwise go undetected.

In cases where ultrasound is inconclusive, Monti et al. highlight the complementary value of PET and MRI, particularly for monitoring large-vessel involvement. These imaging techniques are essential in capturing ongoing inflammation in patients who remain asymptomatic yet have active disease.

## Complications: preventing high-stakes outcomes

Complications from untreated or inadequately managed GCA can be severe, including sight loss, stroke, and aortic aneurysms. Ayo-Martin et al. introduced a novel method of using vertebral artery diameter measured via ultrasound to indicate vertebral vasculitis, which is associated with an increased risk of stroke.

Brekke et al. also highlighted traditional cardiovascular risk factors such as age, smoking, and hypertension as the strongest predictors of mortality in GCA patients. Interestingly, their long-term study found no significant increase in cancer incidence among GCA patients, suggesting that routine cancer screening may not be necessary.

## Mental health in GCA: an overlooked burden

Beyond its physical complications, GCA imposes a significant psychological burden. In a cross-sectional study by Froehlich et al., 40% of GCA patients were found to suffer from major depressive disorder. The study also found a strong correlation between elevated CRP levels and depressive symptoms, suggesting that systemic inflammation may contribute to mental health impairment in GCA patients. These findings underscore the importance of integrating mental health assessments into the routine care of GCA patients, especially considering the potential link between inflammatory markers and psychological distress.

## Steroid-sparing therapies

While glucocorticoids remain the cornerstone of GCA treatment, their long-term use is associated with significant side effects, including osteoporosis, diabetes, and increased infection risk. Biologic therapies, such as tocilizumab, offer a targeted approach to reducing inflammation while sparing patients from glucocorticoid-related side effects. Pankow et al. reported promising results using mycophenolate mofetil (MMF) as a steroid-sparing therapy for GCA, significantly reducing CRP levels and disease remission in a small cohort. These findings pave the way for future randomized controlled trials to establish MMF's role in the broader treatment landscape for GCA.

In addition, glucocorticoids reduce the sensitivity of various diagnostic methods. Stamatis et al. emphasize that TAB should ideally be performed before initiating glucocorticoids to preserve diagnostic accuracy.

## Conclusion: a path toward precision medicine

GCA remains a complex condition that requires a dynamic approach to diagnosis and management. The combined use of advanced imaging and the GCAS represents a significant step forward in diagnosing, monitoring, and managing GCA. Haaversen et al. and Monti et al. emphasize the importance of a multimodal approach that includes imaging to track disease progression and detect relapses. The use of steroid-sparing therapies such as mycophenolate mofetil and the focus on personalized care is transforming the landscape of GCA. In the future, treatment will be tailored to each patient's unique disease characteristics, ensuring optimal outcomes while minimizing complications.

